# Endoscopic management of low output recurrent colonic fistula or leak after anterior resection for rectal cancer: a randomized controlled trial

**DOI:** 10.1007/s00464-023-10092-z

**Published:** 2023-05-05

**Authors:** Said Negm, Ahmed Farag, Ahmed Shafiq, Ehab Abd Allah, Mohamed Shehata, Yasser A. Orban, Mohamed Saleh, Amr A. Abdelghani

**Affiliations:** General Surgery, Faculty of Medicine, Zagazig University, Zagazig, Egypt

**Keywords:** Colonic fistula, Endoscopy, Leak, Cancer Rectum, Endo-clip

## Abstract

**Background:**

Colonic anastomotic leak and fistula following anterior resection surgery for rectal cancer are associated with high mortality rates. The incidence of occurrence varies from 2 to 25% and it is difficult to accurately calculate the incidence of fistula and leak post anterior resection, as most of them are asymptomatic. Endoscopic management of fistula and leak has become the first line of management after conservative management in many gastrointestinal surgical centers with the advantages of being less invasive, shorter length of post-operative hospital stay, effective and rapid recovery in comparison to revision surgery. Effective endoscopic management for colonic fistula or leak depends on the clinical status of the patient and fistula characters (time-to-occur and size and site of defect), and device availability.

**Methods:**

This prospective randomized controlled clinical trial included all patients who developed the manifestations of low output recurrent colonic fistula or leak after colonic anterior resection for rectal cancer at Zagazig University Hospital between (December 2020 and August 2022). Sample size was 78 patients divided into two equal groups. Endoscopic group (EG): included 39 patients who underwent endoscopic management. Surgical group (SG): included 39 patients who underwent surgical management.

**Results:**

The investigators randomized eligible 78 patients into two groups: 39 patients in SG and 39 patients in EG. The median size of the fistula or leak was nine (range: 7–14) mm in EG, versus ten (range: 7–12) mm in SG. Clipping and Endo-stitch device were used in 24 patients versus 15 patients, respectively, in EG while primary repair with ileostomy, and resection & anastomosis were used in 15 patients versus 24 patients, respectively, in SG. Recurrence, abdominal collection, and mortality were the post procedure’s complications with incidence of occurrence of 10.3, 7.7 and 0%, respectively, in EG versus 20.5, 20.5 and 2.6%, respectively, in SG. Excellent, good, and poor were the parameters for quality of life with incidence of occurrence of 43.6, 54.6 and 0%, respectively, in EG versus 28.2, 33.3 and 38.5%, respectively, in SG. Median hospital stay was one (range: 1–2) day in endoscopic group, and seven (range: 6–8) days in SG.

**Conclusion:**

Endoscopic intervention may offer a successful modality in managing low output recurrent colonic fistula or leak after anterior resection for rectal cancer that did not respond to conservative measures in stable patients.

ClinicalTrials.gov ID: NCT05659446.

Anastomotic leak and colonic fistula following anterior resection surgery for rectal cancer are associated with high mortality rates [1]. Although, the reported incidence rate varies from 2 to 25%, it is difficult to accurately count the incidence rate of fistula and leak post anterior resection because most of them are asymptomatic [2]. Fistula is defined as abnormal tract between two epithelized surfaces and can be a complication of leak due to acute or chronic inflammation process [3] while leak is defined as a pathological connection between intra & extra body compartments due to anastomotic defect, and chronic leak may lead to fistula between two epithelized surfaces [4]. The classification of fistula depends on many parameters, one of them depends on fistula's output and if it is below 500 cc/day, the fistula is classified as a low output fistula and can be managed conservatively or endoscopically [5] while the role of revision surgery may be reserved for high output colonic fistula with sepsis [6]. In most of gastrointestinal centers, endoscopic management of fistula and leak has become first line of management following failure of conservative management. The endoscopic approach is characterized by less invasiveness, shorter length of post-operative hospital stays and effective and rapid recovery, when compared to revision surgery [7]. Effective endoscopic management for colonic fistula or leak depends on the clinical status of the patient, onset, time, size and site of defect, and device availability [8]. Clips & Endo-stents are the most commonly available endoscopic devices used for management of colonic fistula or leak, but there are also many devices such as endoscopic internal drainage, Endo-suturing and vacuum system [9]. In this study, we will focus on the effect of clipping and Endo-suturing vs open surgical management for treating of low output colonic fistula or leak after anterior resection operation for rectal cancer.

## Objectives

To evaluate the advantages and safety of endoscopic management vs open surgical management of low output recurrent colonic fistula or leak after anterior resection for rectal cancer.

### Patients and methods

#### Patients

This prospective randomized controlled clinical trial included all patients who developed the manifestations of low output recurrent colonic fistula or leak after colonic anterior resection for rectal cancer at Zagazig University hospital between (December 2020 and August 2022). The study was prospectively approved by Zagazig University Faculty of Medicine Institutional Review Board (Approval Number: 10027/26-10-2022) and was retrospectively submitted in clinical trials.gov in November 2022 (ClinicalTrials.gov ID: NCT05659446). The investigators performed the study under the code of ethics of the World Medical Association (Declaration of Helsinki) for studies involving human subjects. Written informed consent was obtained from all study participants. Patients with recurrent low output colo-cutaneous fistula (less than 500 cc/24 h) or leak after anterior resection due to rectal cancer, patients with unsuccessful conservative measures, patient with good general condition (ASA I and II), patients with size of fistula less than 15 mm and patients with good nutritional status were included and eligible for randomization. The investigators excluded patients who were in bad general condition (ASAIII&IV&V), patients with high output fistula, patients with recto-vaginal or recto-vesical fistula, patients with size of fistula more than 15 mm or patients who were successfully managed with conservative measures.

Included eligible patients were simply randomized at a 1:1 ratio to “Endoscopic (EG)” or “Surgical Group (SG)” via the drawing of sealed envelopes containing computer-generated random numbers prepared by a third party before the start of the intervention.

The sample size was calculated using an open Epi program depending on the following data; confidence interval 95%, power of the test 80%, ratio of unexposed/ exposed 1, the success rate of endoscopic management of low output colonic fistula after anterior resection of rectal cancer versus surgical management was 60.2% versus 90%, respectively. Odd ratio 0.17, and risk ratio 0.67, so the calculated sample size equal 78 patients divided into two equal groups.

Primary and secondary outcomes were success rate of managing the fistula, length of postoperative hospital stay, and complications and mortality in each group after the intervention during the 3-months follow-up period, respectively. The clinical data of the patients was stored in hard drive that was protected by a password.

#### Diagnosis

After full history taking and complete physical examination, low output colonic fistula or leak after anterior resection for rectal cancer was clinically suspected and then confirmed by laboratory investigations (complete blood picture, liver and kidney functions, coagulation profile), radiological imaging (abdominal US to exclude any abdominal collection, CT abdomen with oral and I. V contrast, MRI in some situations, and virtual colonoscopy to exclude any distal obstruction, distal narrowing or recurrence of the cancer).

#### Intervention

The main concept for management of leak and fistula is draining the leaked gastrointestinal content (decontamination) that can be achieved surgically or endoscopically. We start with general measures including: bowel rest, intravenous fluid when clinically indicated, antibiotic coverage, nutritional status improvement, drainage of any peritoneal collection by Interventional Radiology, and hemodynamic monitoring.

Patients involved in EG were firstly subjected to Interventional Radiology to drain any intra-peritoneal collection diagnosed by preoperative radiology, then were subjected either to Clips application (OTSC, OVASCO Endoscopy AG. Tubingen, Germany) or Endo-suturing (Overstitch, Apollo Endo-Surgery, TX, United States) to close the low output fistula or leak after anterior resection for rectal cancer. After colonic preparation (chemical & mechanical preparation), the endoscopic procedure was performed under sedation (no general anesthesia) in order to detect the size of fistula. Clips were used in cases with fistula's size was less than 10 mm, while Endo-suturing devices were used in cases with fistula's size more than 10 mm and up to 15 mm.

Patients involved in SG were subjected to either open redo of resection anastomosis manually, by a circular stapler or primary repair of the defect with ileostomy. This was performed under general anesthesia after colonic preparation. No patients underwent temporary ileostomy at time of primary surgery for rectal cancer.

#### Follow up after endoscopy and discharge from the hospital

All patients were subjected to clinical examination & laboratory investigations during the hospital stay. Any suspected colonic leak or fistula post intervention mandated a CT scan with oral and I. V contrast and lower GI endoscopy. Patients were followed-up for at least 3-months post repair.

#### Statistical analysis

Analysis of data were done by IBM computer using SPSS (statistical program for social science version 23) as follows: Description of quantitative variables as Mean, SD, median and IQR, Shapiro test of normality used to check the data distribution, Description of qualitative variables as number and percentage, Chi-square test was used to compare qualitative variables between groups, Fisher exact test was used when one expected cell or more are less than 5, and Mann–Whitney test was used instead of unpaired *t*-test in non-parametric data (SD > 30% mean). *P* value > 0.05 is considered insignificant, while *P* < 0.05 is considered significant [10].

## Results

**Fig. 1:**
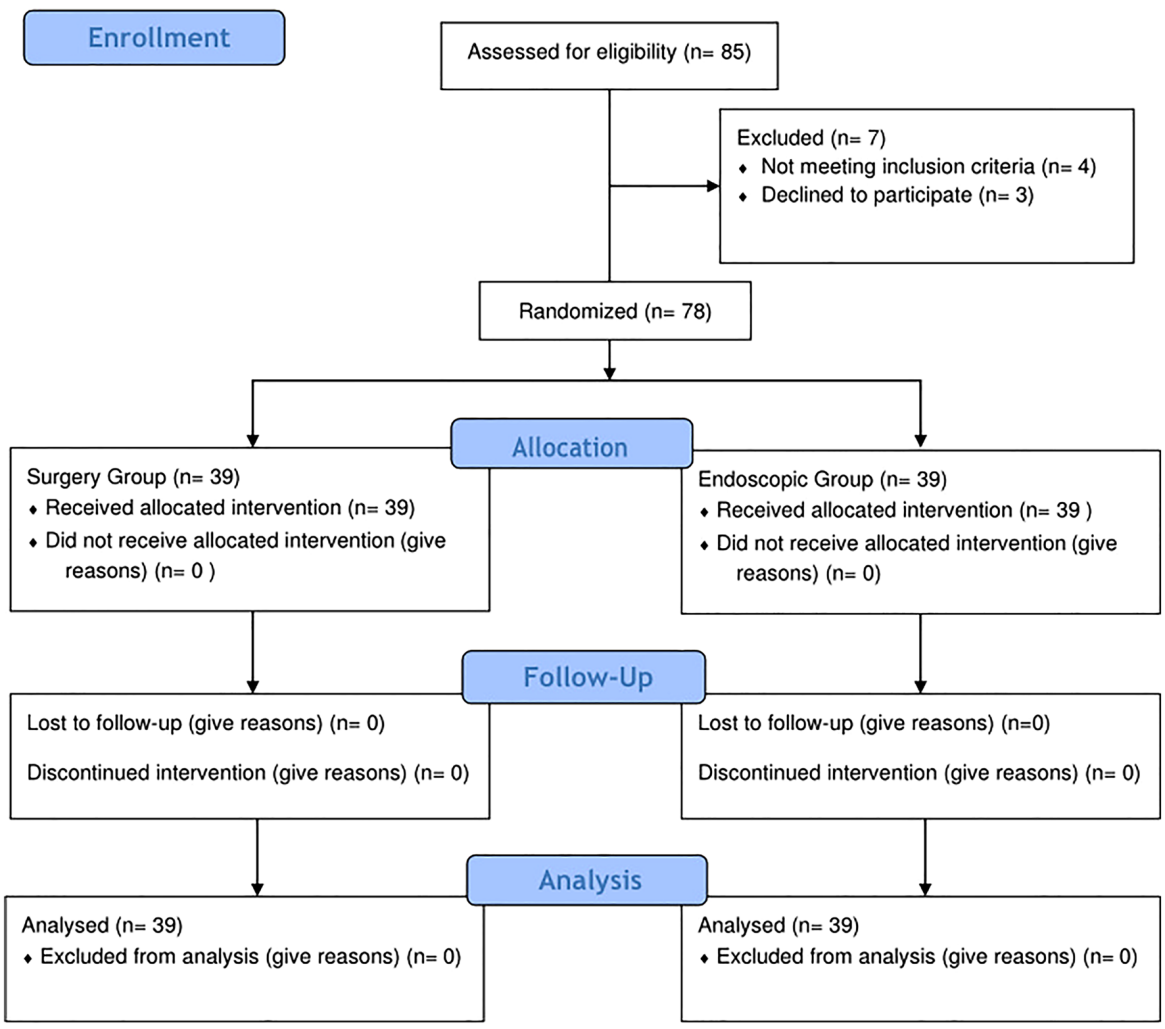
CONSORT flow diagram

Out of 85 patients, only 78 patients were eligible for this study. Seven patients were excluded (three patients were successfully managed conservatively, one patient developed septic shock, and the other three patients refused to participate in the study). Therefore, the final sample size was 78 patients divided into two equal groups. EG: included 39 patients and SG included 39 patients (Fig. 1).

Males in both groups constituted 67.90% (58/78) of the patients. The mean age in both groups was 57.64 ± 16.59 years-old. All patients in both groups were subjected to neoadjuvant therapy. The co-morbidities among patients of both groups were diabetes mellitus, hypertension and obesity with incidence rates of 23.10, 43.60, and 10.30%, respectively. The mean time-to-fistula development postoperatively in both groups was 9.4 ± 2.74 days. Staging of rectal cancer in EG was 14 (35.8%) patients with stage 1, 20 (51.2%) patients with stage II and 5 (12.8%) patients with stage III while in surgical group was 12 (30.7%) patients with stage I, 22 (56.45) patients with stage II and 5 (12.8%) patients with stage III. In both groups, anterior resection, low anterior resection, and ultra-low anterior resection were the primary surgery for rectal cancer in 75.60, 11.50, and 12.80% of patients, respectively. The mean hemoglobin level among patients in both groups was 11.06 ± 1.82 mg/dl versus mean albumin level of 3.77 ± 0.71 g. All patients in EG were subjected to endoscopy without the need for general anesthesia, while all the patients in SG needed anesthesia. The mean size of the fistula was 9.86 ± 3.47 mm among the patients in both groups. In EG, Clipping & Endo-stitch device were used in 24 and15 patients, respectively). In SG, primary repair of fistula with ileostomy & resection anastomosis were used in 16 and24 patients, respectively. The incidence rates of reported recurrence of fistula, abdominal collection and mortality were 15.40, 14.10 and1.30%, respectively) among the patients in both groups. The quality of life in both groups was reported as excellent, good and poor in 35.90, 44.90 and 19.20%, respectively among the patients in both groups. Median length of hospital stay was one (range:1–2) day in EG while in SG was seven (range: 6–8) days.

Comparison of qualitative variables between both groups revealed (Tables 1, 2), males constitute 27 69.2% (27) of the patients EG versus 26 66.7% (26) of the patients in SG. DM, HPN, obesity were the comorbidities with incidence rates of 23.1, 30.8 and 12.8%, respectively, in EG versus 23.1, 56.4, and 7.7%, respectively, in SG. Anterior resection, low anterior resection and ultra-low anterior resection were the primary surgical procedures performed for rectal cancer in 84.6, 7.7, 7.7% of patients in EG versus 66.7, 15.4, 17.9% in SG. The median time-to-fistula occurrence was nine (range: 7–11) days in EG versus ten (range: 7–12) days in SG. The median hemoglobin level in EG was 11 (range: 10–13) mg/dl versus 10 (range: 9–12) mg/dl in SG. The median albumin level was 4 (range:3.2–4.5) gm in EG versus 3.5 (range: 3–4) gm in SG. The median size of the fistula or leak was 9 (range:7–14) mm in EG, versus 10 (range: 7–12) mm in SG. Clipping and Endo-stitch device were used in 24 patients and 15 patients in EG, respectively, while primary repair with ileostomy, and resection and anastomosis were used in 15 and 24 patients in SG, respectively. Recurrence, abdominal collection and mortality were the post procedures complications with incidence rates of 10.3, 7.7 and 0%, respectively, in EG versus 20.5, 20.5 and 2.6%, respectively, in SG. Excellent, good and poor scores were the parameters for quality of life with incidence rates of 43.6, 56.4.6 and 0%, respectively, in EG versus 28.2, 33.3 and 38.5%, respectively, in SG.

**Table 1:** Comparison of different factors (Sex, comorbidities, tumour staging, types of primary surgery, need for general anaesthesia, type of intervention, complications and quality of life) between endoscopy and surgical groups

	Endoscopy group	Surgical group	*P* value	Statistical test
	N (%)	N (%)		
Sex				
Male	27(69.2)	26(66.7)	0.808	Chis quare
Female	12(30.8)	13(33.3)		
Co-morbidities				
DM	9(23.1)	9(23.1)	1	Chis quare
Hypertension	12(30.8)	22(56.4)	0.022	Chis quare
Obesity	5(12.8)	3(7.7)	*0.711	
Tumour staging				
Stage I	14(35.8)	12(30.7)	0.88	Chis quare
Stage II	20(51.2)	22(56.4)	0.88	Chis quare
Stage III	5(12.8)	5(12.8)	0.88	Chis quare
Type of primary surgery				
Anterior resection	33(84.6)	26(66.7)	0.065	Chis quare
Low anterior resection	3(7.7)	6(15.4)	0.481	Chis quare
Ultralow anterior resection	3(7.7)	7(17.9)	0.176	Chis quare
Needs for general anaesthesia				
No	39(100)	0(0)	< 0.001	Chis quare
Yes	0(0)	39(100)		
Type of intervention				
Clipping	24(61.5)	0(0)	< 0.001	Chis quare
Endo-stiching	15(38.5)	0(0)	< 0.001	Chis quare
Primary with ileostomy repair	0(0)	15(38.5)	< 0.001	Chis quare
Resection &amp; anastomosis	0(0)	24(61.5)	< 0.001	Chis quare
Complications				
Recurrence	4(10.3)	8(20.5)	0.209	Chis quare
Abdominal collection	3(7.7)	8(20.5)	0.104	Chis quare
Mortality	0(0)	1(2.6)	1	Chis quare
Quality of life				
Excellent	17(43.6)	11(28.2)	0.157	Chis quare
Good	22(56.4)	13(33.3)	0.04	Chis quare
Poor	0(0)	15(38.5)	< 0.001	Chis quare

**Table 2:** Comparison of different factors (age, Hb level, Albumin, Timing of fistula, Size of fistula, hospital stay) between endoscopy and surgical groups

	Endoscopy Group	Surgical Group	*p* value
	Median (IQR)	Median (IQR)	
Age (years)	60(43:70)	60(45:75)	0.568
Hb level (mg/dl)	11(10:13)	10(9:12)	0.169
Albumin (g/dl)	4(3.2:4.5)	3.5(3:4)	0.058
Timing of fistula in days	9(7:11)	10(7:12)	0.209
Hospital stay in days	1(1–2)	7(6–8)	0.00001
Size of fistula in mm	9(7:14)	10(7:12)	0.944

Mann Whitney test of Significant

In Multiple Logistic regression models conducted to find significant predictors to good quality of life, the significant predictors were endoscopy group and not diabetic patient as seen in Tables 3, 4. In univariable analysis, the test of significance between excellent & poor qualities of life among the patients in both groups was not significant, while there was a statistically significant difference between good and poor qualities of life among patients in both groups, so in regression model we took the significant results only.

**Table 3:** Factors associated with good quality of life in studied group

		poor quality of life	Good quality of life	*p* value
		N (%)	N (%)	
Group	Endoscopy Group	0(0)	22(62.9)	< 0.001
	Surgical Group	15(100)	13(37.1)	
Sex	Male	11(73.3)	22(62.9)	0.474
	Female	4(26.7)	13(37.1)	
Comorbidities				
DM	No	6(40)	29(82.9)	0.006*
	Yes	9(60)	6(17.1)	
HTN	No	6(40)	22(62.9)	0.136
	Yes	9(60)	13(37.1)	
Obesity	No	12(80)	30(85.7)	0.614
	Yes	3(20)	5(14.3)	
Complications				
Recurrence	No	7(46.7)	31(88.6)	0.003*
	Yes	8(53.3)	4(11.4)	
Abdominal collection	No	7(46.7)	32(91.4)	< 0.001
	Yes	8(53.3)	3(8.6)

**Table 4:** Multiple regression analysis for quality of life

	*p* value	OR	95% C.I. for OR	
			Lower	Upper
Age years	0.062	1.064	0.997	1.137
Diabetes(yes/no)	0.035	19.008	1.23	293.673
Complications recurrence (yes/no)	0.134	6.932	0.549	87.466
Intervention (Endoscopy group/ surgical group)	0.003	125.104	5.433	2880.942
Constant	0.01	0.001		

*OR* odds ratio, *95% C.I.* confidence interval. Multiple Logistic regression conducted to find significant predictors to good quality of life. The significant predictors was endoscopy group and not diabetic patient

## Discussion

Firstly, there is great deference in terms of perforation, leak and fistula. Perforation means acute full thickness (transmural) defect in the gastrointestinal tract, occurring either spontaneously or due to trauma (after an injury and iatrogenic) [11]. Leak means disruption of gastrointestinal anastomosis that leads to fluid collection, and the site of leak is usually related to the site of the anastomosis and can be intra-or extra-peritoneal [12]. Fistula means an abnormal tract between two epithelized surfaces and can be internal (Entero-Enteric fistula) or external (Entero-Cutaneous) [13].

There is an algorithm for management of leak and fistula. Regarding the fistula management, drainage of any collection by interventional radiology with concurrent antibiotics & nutritional status improvement are the first steps, then the management depends on the size of fistula: (a) if the size is more than 2 cm, it needs fistulotomy; (b) if size is less than 2 cm, attempts of primary closure should be tried; (c)if size of fistula is less than 10 mm, the OTSC is used; (d) if the size of fistula more than 10 mm but less than 2 cm, Endo-stitch devise is used [14].

For leak, the management algorithm depends on the clinical status for the patient: (a) if patient presents with unstable clinical condition, he will be subjected for surgery; (b) if presenting with stable clinical condition, the patient will be subjected for endoscopic management according to size of the leak: (b.1) if the size is more than 2 cm, it needs resection & anastomosis; (b.2) if the size is less than 2 cm, attempts of primary closure should be tried; (b.3) if the size of fistula is less than 10 mm, the OTSC is used; (b.4) if the size of fistula is more than 10 mm but less than 2 cm, Endo-stitch device is used [14, 15].

In this study, for closure of low output fistula or colonic defect with size of less than 1.5 cm, the investigators used OTSC (over-the-scope clips) and Endo-suturing device. OTSC was used to close colonic defect or fistula with size of less than 1 cm. The investigators in this study used OTSC with no evidence of clip migration or postoperative stricture during the postoperative follow-up period [16]. This type of Endo-clips is used to close full thickness defect unlike another type of Endo-clips that are called TTSCs (over-the-scope clips) that are used to close only mucosa and sub-mucosa. OTSCs are applied in perpendicular manner to the long axis of the fistula or defect after thermal ablation or mechanical scratching of the tissue around the defect to achieve a good result in sealing the defect [17]. More than one OTSC can be used to close the defect, starting from the periphery of the defect to its center [18]. Endo-suturing device is used to close the defect with size ranges from 10 to 15 mm, with the advantage of full thickness closure of the wall defect but needs high experience. The Apollo Overstitch (Apollo Endo-surgery, Austin, TX, USA) is a non-reusable device allowing continuous or intermittent suturing with a cinching tool. The device is loaded frontally onto a double channel endoscope [19]. The main advantage of Apollo Overstitch is that it can be reloaded inside the body without the need for removing it between stitches and permits one endoscopic channel to be free [20]. The maneuver is started with de-epithelialization of the edges of the defect using argon plasma coagulation before the Overstitch system application [21].

There are many advantages of endoscopy that made it a cornerstone in managing such type of fistula & leak like shorter hospital stay, no abdominal scar, less pain, low incidence of surgical trauma, early return to normal active life, low incidence of infection, rapid recovery, and low incidence of nerve damage.

Regarding post-operative complications in the endoscopic group, recurrence of fistula occurred in four patients due to lack of adequate experience, inadequate drainage of peritoneal collections, recurrence of primary tumor due to delay in postoperative adjuvant chemo-radiotherapy due to delay in management of fistula and inadequate nutrition. Abdominal collection in this group (EG) occurred in three patients due to recurrence of fistula or leak and inadequate drainage of peritoneal collections. In the surgical group, recurrence occurred in 8 patients due to inadequate surgical repair, recurrence of primary tumor due to delay in postoperative adjuvant chemo-radiotherapy and poor nutritional status. Abdominal collection in this group (SG) occurred in 8 patients due to recurrence of fistula or leak and inadequate drainage of peritoneal collections. In Surgery Group, one patient died due to recurrence of fistula or leak with uncontrolled sepsis. Staging of the rectal cancer did not affect healing of fistula in both groups as healing of fistula depended mainly on proper control of sepsis, improvement of nutritional status, absence of distal obstruction, surgeon's experience, no tumor recurrence and adequate safety margin of primary surgery.

Regarding quality of life, we used WHOQOL-BREF questionnaire that has 26 items. This questionnaire has four domains included physical health (seven items), psychological (six items), social relationship (three items) and environmental health (eight items); it also has QOL and general health items. Each separate item is scored from 1 to 5 on a response scale. The scale is then stipulated as a five point ordinal scale. The score is then changed linearly to a 0–100 scale. The physical health domain includes items on mobility, daily activities, functional capacity, energy, pain, and sleep. The psychological domain includes items on self-image, negative thoughts, positive attitude, self-esteem, mentality, learning ability, memory concentration, religion, and mental status. The social relationship domain includes items on personal relationship, social support, and sex life. The environmental heath includes items on financial resources, safety, health and social services, living physical environment, opportunities to acquire new skills and knowledge, recreation, general environment (noise, air pollution, etc.), and transportation. The score below 50 means poor quality of life, score between 50 to 75 means good quality of life and score more than 75 means excellent or high quality of life [22]. In our study, the quality of life of the patients in endoscopic group is better than the quality of life of patients in surgical group. In Multiple Logistic regressions conducted to find significant predictors to good quality of life. The significant predictors were endoscopy group and not diabetic patient.

The main limitations of this study are small sample size, exclusion of patients with ASA III and IV, short follow-up period, large sized defect more than 2 cm can't be managed by clipping or Endo-stitch and de-epithelization of the edge of the defect that was done by argon plasma or mechanical scratching depended on the endoscopist own experience. However, the point of strength of this study was being of randomized clinical trial comparing different modalities of the endoscopic approach on one hand and surgical approach on the other hand [23].

## Conclusion

Endoscopic intervention can be a successful modality in managing low output recurrent colonic fistula or leak after anterior resection for rectal cancer without the need for surgical intervention. No recurrence of leak or fistula was noted after endoscopic clipping or stitching. Further studies with large sample size and longer follow-up period are required to conclude strong and valid results.

## Data Availability

All data generated during this study are included in this published article and its supplementary information files. Further minor datasets are available from the corresponding author on reasonable request.

## Abbreviations

EG: Endoscopic group
SG: Surgical group
DM: Diabetes mellitus
HPN: Hypertension
IHD: Ischemic heart disease
BMI: Body mass index
